# Differences in the Association between Segment and Language: Early Bilinguals Pattern with Monolinguals and Are Less Accurate than Late Bilinguals

**DOI:** 10.3389/fpsyg.2016.00993

**Published:** 2016-06-29

**Authors:** Cynthia P. Blanco, Colin Bannard, Rajka Smiljanic

**Affiliations:** ^1^Department of Linguistics, University of Texas at AustinAustin, TX, USA; ^2^Department of Psychological Sciences, University of LiverpoolLiverpool, UK

**Keywords:** speech perception, foreign-accented speech, bilingualism, language categorization, Spanish phonology, English phonology, metalinguistic awareness

## Abstract

Early bilinguals often show as much sensitivity to L2-specific contrasts as monolingual speakers of the L2, but most work on cross-language speech perception has focused on isolated segments, and typically only on neighboring vowels or stop contrasts. In tasks that include sounds in context, listeners’ success is more variable, so segment discrimination in isolation may not adequately represent the phonetic detail in stored representations. The current study explores the relationship between language experience and sensitivity to segmental cues in context by comparing the categorization patterns of monolingual English listeners and early and late Spanish–English bilinguals. Participants categorized nonce words containing different classes of English- and Spanish-specific sounds as being more English-like or more Spanish-like; target segments included phonemic cues, cues for which there is no analogous sound in the other language, or phonetic cues, cues for which English and Spanish share the category but for which each language varies in its phonetic implementation. Listeners’ language categorization accuracy and reaction times were analyzed. Our results reveal a largely uniform categorization pattern across listener groups: Spanish cues were categorized more accurately than English cues, and phonemic cues were easier for listeners to categorize than phonetic cues. There were no differences in the sensitivity of monolinguals and early bilinguals to language-specific cues, suggesting that the early bilinguals’ exposure to Spanish did not fundamentally change their representations of English phonology. However, neither did the early bilinguals show more sensitivity than the monolinguals to Spanish sounds. The late bilinguals however, were significantly more accurate than either of the other groups. These findings indicate that listeners with varying exposure to English and Spanish are able to use language-specific cues in a nonce-word language categorization task. Differences in how, and not only when, a language was acquired may influence listener sensitivity to more difficult cues, and the advantage for phonemic cues may reflect the greater salience of categories unique to each language. Implications for foreign-accent categorization and cross-language speech perception are discussed, and future directions are outlined to better understand how salience varies across language-specific phonemic and phonetic cues.

## Introduction

Listeners make judgments about talkers and their speech after only brief exposure. Considerable work has investigated the suprasegmental and segmental acoustic cues most important for listeners in their decisions about talker-specific characteristics like region of origin, age, and gender (Klatt and Klatt, 1990; Strand and Johnson, 1996; Harnsberger et al., 1997; Strand, 1999; Clopper and Pisoni, 2004, 2007; Tracy et al., 2015). Other cues may indicate that a talker grew up using a language other than the one being spoken, yielding a foreign accent (e.g., Flege, 1991; Flege and Munro, 1994; Flege et al., 1997a,b). At times it may even be necessary for listeners to identify which language a talker is using, for example, so that a bilingual can map a new word to the appropriate language or to facilitate a bilingual’s access of a known word in one of their languages (Flege, 2007). However, unlike the work investigating associations of acoustic properties with indexical information like region of origin, cross-language speech perception tasks typically test only isolated vowels without a larger phonological context or consonants in a single CV syllable (although some work also presents stop bursts without context, e.g., Flege, 1984). These segments are often very limited in range (e.g., comparing neighboring vowels only). It is therefore unclear which segmental cues are most useful to listeners in making distinctions between their languages or whether listeners attend to all language-specific acoustic cues equally. The current project seeks to test listener sensitivity to a range of language-specific segments in nonce word contexts and considers how a listener’s language background influences their use of these cues in a cross-language speech perception task.

Previous work has examined how listeners’ language experience shapes their ability to categorize or discriminate isolated, or nearly-isolated, segments and subsegmental cues in cross-language speech perception. In these studies, bilingual listeners categorize or discriminate between pairs or triplets of sounds ranging along a continuum, most often the VOT continuum (e.g., between /t/ and /d/) or formant continua between neighboring vowels in the L2 (e.g., /i/ and /I/). These studies have shown that monolingual English listeners and early bilinguals make similar distinctions among English categories (e.g., Mack, 1989; Flege et al., 1999a), and that this is especially true for bilinguals who have lower rates of continued use of or exposure to their L1 (Flege and MacKay, 2004). In some vowel discrimination tasks, even late bilinguals pattern like English monolinguals (Flege et al., 1994). However, listeners use a host of cues when perceiving speech beyond isolated segments or syllables, and in fact, differentiating native and non-native stop bursts may not require accessing linguistic representations at all, as is the case when listeners make parallel judgments between continua of non-speech sounds (Pisoni, 1977; Diehl and Walsh, 1989). It is possible that listeners use different, even non-linguistic and general auditory, strategies to make decisions about the isolated segments and syllables and acoustic cues used in these identification and discrimination tasks (Flege, 1987). Furthermore, these studies typically only evaluate listener sensitivity to cues in the L2, most often English, so very little is known about how they process segments particular to their first language.^1^

A few studies have attempted to extend the findings on the perception of segments in isolation or in syllables to the perception of language-specific speech and accented productions in longer stimuli. In a series of experiments, Flege (1984) found that listeners could distinguish native and non-native talkers of English after hearing CV syllables, single words, and three-word phrases. Even more remarkably, native English listeners could use input as brief as 30 ms of a stop burst to differentiate productions from native- and French-accented talkers. However, it is not clear that the strategies listeners used are the same across these varying materials despite the fact that listeners mostly accurately categorized stimuli from across this range of input. For the longer utterances, listeners may not have necessarily made use of stop burst differences at all, even though they can identify these differences in other tasks. Instead, listeners may pay more attention to other segmental and suprasegmental cues present in the longer stretches of speech. That is, the presence of a usable language-specific cue like a stop burst does not necessarily mean that this will be the most useful cue when other cues are present, and other cues may in fact be more salient to listeners than VOT. For example, evidence from a perceptual-similarity task using phrase-length stimuli from 17 languages suggests that marked back consonants and front vowel rounding might be particularly salient dimensions for non-native listeners (Bradlow et al., 2010). However, there remains some question about the interpretation of at least the vowel dimension in the perceptual-similarity study, so the number of cues present in even short phrases makes it difficult to identify the most influential acoustic factors.

Flege and Munro (1994) tested listener sensitivity to the multiple cues available in word-length stimuli by asking monolingual English listeners to categorize productions of *taco* as having been produced in English or in Spanish. The length of VOT associated with the initial /t/ explained more variance in listeners’ responses than any other acoustic cue, but this language-specific difference is confounded with having occurred so early in the word – listeners may not have attended to the whole word if they could confidently make a decision based on the first segment or syllable. Since all four segments were Spanish-like or English-like in any production of *taco*, the results also do not reveal which cue(s) listeners would rely on, in the absence of the other cues. The VOT of /t/ was the strongest cue, but it is unclear if the other cues would have been sufficient for listeners to categorize productions accurately. The sensitivity of monolingual listeners to language-specific stops in Flege (1984) and Flege and Munro (1994) suggests that listeners can compare the VOT of the stimulus to their stored representations of what is an acceptable or atypical VOT for English stops. It remains to be seen whether bilinguals would show the same sensitivity to these cues in more naturalistic, word-length contexts. By manipulating a single cue in a stimulus word, and holding constant the remaining segments, we can begin to understand whether listeners from different language backgrounds can make use of a given cue when evaluating their lexical representations.

Work from mispronunciation studies indicates that bilingual listeners who can easily discriminate segments or syllables in isolation might be less able to identify those same differences in word-length stimuli, and this disparity across tasks is true even for early, highly proficient bilinguals. Listeners in these studies complete identification and discrimination tasks, and then identify whether a stimulus is the typical pronunciation of the word or if it is mispronounced. For the segment identification tasks contrasting neighboring vowels in Catalan (e.g., /ε/∼/e/), there are conflicting results: highly proficient Spanish-dominant Spanish–Catalan bilinguals in Barcelona were unable to reliably distinguish the Catalan mid-vowels is isolation (Sebastián-Gallés and Soto-Faraco, 1999), while their peers in Majorca were successful (Amengual, 2015). However, Spanish-dominant bilinguals in both locales responded similarly poorly in the mispronunciation tasks, in which they heard a word’s actual mid-vowel replaced with the neighboring vowel (e.g., /ε/ replaced with /e/, as in /Әrεl/ ‘root’ pronounced as ^∗^/Әrel/). Sebastián-Gallés and Soto-Faraco (1999) and Sebastián-Gallés et al. (2005) attribute the lack of detail in Spanish-dominant bilinguals’ representations of Catalan to their exposure to Spanish in the first years of life, before acquiring Catalan. However, Amengual’s results indicate that early Spanish exposure itself is not the cause of early bilinguals’ decreased discrimination abilities in the mispronunciation task, since listeners in Majorca could reliably perceive differences when the segments were presented in isolation. This suggests that, in both regions, the Spanish-dominant bilinguals’ lexical representations of Catalan contain less phonetic detail for Catalan-specific contrasts, despite the ability of some listeners to discriminate the segments in other tasks. This difference in the detail of bilinguals’ lexical representations reflects the kinds of variation to which listeners are exposed, and the construction of representations is likely more complex than would be suggested by a listener’s ability to discriminate isolated sounds or syllables. It is therefore important that investigations into the nature of bilinguals’ representations of their languages use tasks that force listeners to respond to more complex input as language to better understand the level of detail encoded in lexical representations and to more closely approximate the challenge of processing naturalistic speech.

In fact, lexical representations incorporate not only phonological variation but social information associated with that variation as well. These indexical features, such as speaker and contextual characteristics, are encoded in the lexical representations, and they may be incorporated even after only brief exposure in the lab (e.g., Nygaard and Pisoni, 1998; Allen and Miller, 2004; Kraljic and Samuel, 2006, 2007). If the Spanish–Catalan bilinguals heard more variable input in the productions of real words, their representations of Catalan may have included both productions as possible, explaining their difficulty identifying mispronunciations, whereas the monolinguals in Flege (1984) and Flege and Munro (1994) may have been exposed to less variation in English and so were more sensitive to deviations from typical productions. There is also evidence demonstrating that listeners with exposure to specific accents, even in absence of knowing the L2, show improved processing and categorization of those accents (Clopper and Pisoni, 2004, 2007; Vieru et al., 2011; Witteman et al., 2013), so language and a talker’s language proficiency must also be linked to specific productions.

These associations of indexical information with productions, and the incorporation of acoustic variation in lexical representations, are in line with exemplar theories of speech perception (Johnson, 1997; Pierrehumbert, 2002). Listeners use stored exemplars – those from an exposure period in a lab or from hearing productions in normal life – to inform their expectations about unheard productions and word forms. Thus, listeners can generalize over a number of stored exemplars about what kinds of stops, for example, occur in English or in the productions of a particular talker of English. Listeners like bilinguals who have experience with a sound category in both languages must associate productions with each language in order to make the appropriate conclusions about the phonological categories in each language (as in the related BLINCS model in Shook and Marian, 2013). For example, a Spanish–English bilingual who hears a word produced with a /t/ will store with this exemplar whether the sound was produced in English or Spanish, and information about how it was produced (e.g., the VOT of the stop) will be added to the listener’s representation for the production of /t/ in the language. Spanish–English bilinguals will therefore have developed detailed phonological representations for English and Spanish, and their sensitivity to the distribution of sounds particular to each language might be expected to be greater than that of English monolinguals, who have only English productions on which to base their language representations. While English monolinguals may have some, or even significant, exposure to Spanish-accented English, their knowledge of Spanish phonology will be less than that of bilinguals who have acquired Spanish since birth. In fact, due to existence of multiple (language-specific) categories in the same phonological space, Spanish–English bilinguals’ representations might also be unlike English monolinguals’ in other ways: bilinguals might use categories more extreme than monolinguals to maximize differences between languages (cf. Flege, 1995), or bilinguals’ categories may show evidence of cross-linguistic transfer and be less like the monolinguals’, especially for later-acquired sounds and for later learners (Flege, 2007).

The present study tests the effect of language experience on listener sensitivity to language-specific segments to better understand how language-specific sounds are represented and related in the bilingual lexicon. We use a novel task in which listeners are told they are hearing snippets of continuous speech (either in Spanish or English) and are asked to associate the nonce words containing a Spanish- or English-specific sound with the appropriate language. Accuracy and reaction times (RTs) are compared across listener groups for each of the classes of segment. The use of nonce words has two advantages. First, presenting word-length stimuli forces listeners to process the sounds linguistically and not just auditorily, and there is evidence that listeners in previous studies may have perceived segments without linguistic context differently than when the same sounds were processed as words. Second, unlike real words, nonce-word stimuli avoid inducing lexical effects related to listeners’ actual exposure to the phonological variations of real words. Finally, the use of word-length nonce stimuli, purportedly taken from naturally produced speech, forces listens to generalize over the phonological properties of their languages and decide in which language a given stimulus must have been produced. The present study also extends previous work, which mostly tested contrasts from only one language (e.g., English in Flege’s work and Catalan in the work of Sebastián-Gallés and Amengual), by including cues from both English and Spanish to more fully investigate how listeners’ language backgrounds influence perception in both languages.

The nonce words tested here include segmental categories that are unique to English or Spanish (“phonemic” cues) and segments that vary in how they are implemented phonetically along a continuum between the Spanish variant and the English variant (“phonetic” cues). Similar distinctions among segments have been made for the perception of non-native sounds that vary in similarity to native categories (Best, 1991) and for the acquisition of second language sounds, in the Speech Learning Model (Flege, 1987, 1995). Evidence suggests that sound categories that are “new” to an L2 and have no counterpart in the L1, like the phonemic cues proposed here, are easier to perceive as a distinct category and to produce authentically than “similar” L2 phones that differ along some particular acoustic-articulatory dimension from the L1 variant, like the phonetic cues described here. One study (Flege and Munro, 1994) has specifically examined phonetic cues in context and found that listeners could use these cues to varying degrees depending on the language background of the talker, but no work has directly compared phonemic and phonetic cues. Following Flege and Munro (1994) and the predictions outlined in the Speech Learning Model for new and similar phones, both classes of cues are expected to be successfully associated with their respective languages but phonemic cues are expected to be stronger indicators of language than phonetic cues in a language categorization task.^2^

Finally, this study also systematically compares the sensitivity of monolingual English listeners and early and late Spanish–English bilinguals. Previous work in cross-language speech perception indicates similarities between English monolinguals and early Spanish–English bilinguals in the categorization of English sounds, but evidence regarding how late bilinguals compare to these groups is more limited. It is expected that the bilingual groups will show greater sensitivity to language-specific cues from both languages than the monolinguals, since the bilinguals’ considerable exposure to both English and Spanish productions should foster more reliable associations between language and the phonetic detail in stored representations.

## Materials and Methods

### Materials

#### Language-Specific Target Segments

Three language-specific phonemic cues were chosen for the categorization task: the English-specific segments /θ/ and /ɹ/, and the Spanish-specific trill /r/. We limited the selection of phonemic cues to those sounds that form categories not present in the other language and that do not form a continuum. For example, the English voiced alveolar approximant /ɹ/ and the Spanish voiced alveolar trill /r/ are not different extremes of a continuum between /ɹ/ and /r/, in the way that English and Spanish voiced and voiceless stops vary along a single dimension (VOT). That is, there is not a single dimension or acoustic correlate that distinguishes /ɹ/ and /r/ that could be increased or decreased to derive one from another, since the two sounds are produced with fundamentally different manners of articulation (/ɹ/ as an approximant and /r/ as a trill). One additional English-specific cue was identified for inclusion as a phonemic cue, /θ/. Although /θ/ is a phoneme in Peninsular Spanish (it is produced as /s/ in Latin America), it was included as an English-specific phoneme since exposure to Peninsular Spanish among our listeners was expected to be very limited, and native speakers of Peninsular Spanish were excluded from the study. Early Spanish–English bilingual listeners living in Central Texas, where this study was conducted, may have some exposure to Peninsular Spanish, for example through movies, but are most familiar with Latin American dialects of Spanish. The late bilingual participants likely have more exposure to Peninsular Spanish than early bilinguals, but it is not expected that this exposure would be more influential on L1 representations than native dialect phonology. In fact, many monolingual English listeners probably have exposure to the trill /r/ in Scottish English, also through media, but it would be surprising if their language-segment associations reflected occasional exposure to the trill /r/ in English.^3^ Vowels were excluded as phonemic cues for this language pair for two reasons. First, all five Spanish vowel categories exist in English, minimally in English diphthongs, so there were no Spanish-specific vowels to consider for phonemic cues. Second, English-specific vowels (e.g., /I/) can be differentiated from the nearest shared vowels (e.g., /i/) by both spectral cues and duration differences; while native listeners attend to the spectral differences in these English-specific vowels, non-native listeners may rely on vowel duration to distinguish these categories (Flege et al., 1997a; Escudero, 2006; Kondaurova and Francis, 2008). In this case, non-native listeners would be able to use the duration continuum between the short /I/ and the long /i/. Instead, we wanted to ensure as much as possible that all listener groups included in this study were attending to the same acoustic property of the target segment.^4^

In addition to the phonemic cues, we also tested phonetic cues, which vary along a continuum. These sound categories exist in both languages but their articulation in each language is characterized by sub-phonemic differences in place of articulation. Two language-specific phonetic segments were chosen for the task, the lateral approximant /l/ and the high back vowel /u/. The lateral approximant is produced as a ‘light’ [l] at the alveolar ridge in Spanish, while in American English the segment is realized as the ‘darker’ [ƚ], with an additional closure near the velum, particularly in closed syllables (Recasens, 2004, 2012). The back vowel differs along F2 in English and Spanish: it is fronted to [ʉ] for many speakers of American English and is produced further back, as [u], in Spanish (Mendez, 1982; Bradlow, 1995; Clopper et al., 2005).

#### Nonce Words

Nonce words were created to test the contributions of specific sounds to listeners’ conceptualizations of Spanish and English. All nonce words were disyllabic trochees with either two open syllables (i.e., CVCV) or /l/ in coda position of the first syllable (i.e., CV/l/CV). The CV/l/CV structure was included in the nonce words to provide two phonological contexts for /l/ stimuli that were both permissible in Spanish and in which /l/ was most likely to be velarized to [ƚ] in American English (Recasens, 2012). The inclusion of disyllabic words with stress on the first syllable meant that the second English vowel would be reduced to schwa, resulting in an additional vowel-quality cue beyond the language-specific target segment. However, this strategy was preferred to the development of monosyllabic words for several reasons. Spanish has relatively few monosyllabic words compared to English (cf. Costa and Caramazza, 1999) so monosyllables may be biased toward English responses. The set of possible word-final consonants in Spanish is very small: /ð, s, n, l, Ր/. Some of these are subject to lenition (/ð/) or aspiration (/s/), or are already included as a language-specific target segment (/l/). Words ending in /Ր/ are associated with infinitive morphemes, and /Ր/ is also in free variation with /r/ word-finally. The inclusion of a second syllable and vowel reduction was therefore preferred. Vowel reduction and its potential influence on listeners’ language decisions are addressed in the discussion.

Each nonce word included one language-specific segment that served as a cue to language categorization. The remaining segments in the nonce words exist in both English and Spanish (at least phonemically, as in the case of the English unstressed schwa) and are not expected to differ between the two languages, so that listeners would be obligated to use the target segment for the language categorization decision. The segments identified as common to both English and Spanish were the fricatives /m,f,s,h/^5^ and the affricate /t∫/, which do not differ between the languages in point of articulation or in voicing, and the vowels /i,a/. While /i,a/ are realized somewhat differently in English and Spanish, with the English variants sometimes transcribed as /ij/ and /α/, respectively, these vowels were preferable over others. Mid-vowels are diphthongized in American English, and /u/ was included as a target segment due to the variation in its articulation in English and Spanish. The symbol /i/ is used here to indicate the vowel in Spanish *mi* ‘my’ /mi/ and English *me*, and /a/ is used to represent Spanish *la* /la/ ‘the’ and the vowel in English *cot*. Although /a/ is more variable than /i/ across the languages (Bradlow, 1995), it was included to increase the number of possible stimuli.

For each target segment, eight nonce CVCV and CV/l/CV words were constructed from the set of segments overlapping in English and Spanish. Each nonce word was a possible, but non-existent, word in both English and Spanish, and all words ended with /a/, which was reduced to [Ә] in the English stimuli. See **Table 1** for the set of stimuli containing language-specific phonemes and **Table 2** for the set of stimuli containing language-specific phonetic segments. One phonemic stimulus, *racha*, was identified as a real Spanish word meaning ‘gust of wind’ after the study had been completed, so it was excluded from the following analyses. The English nonce word /ɹat∫Ә/ was also removed due to its similarity to the Spanish *racha* /rat∫a/, since bilingual listeners may have interpreted this stimulus as the Spanish word *racha* produced with an English accent and not as a uniquely English word.

**Table 1:** Nonce words with language-specific phonemes /θ,ɹ,r/.

English phoneme /θ/	English phoneme /ɹ/	Spanish phoneme /r/
/t∫iθӘ/	/t∫aɹӘ/	/t∫ira/


/fiθӘ/	/fiɹӘ/	/fara/


/hiθӘ/	/hiɹӘ/	/fira/


/maθӘ/	/maɹӘ/	/mara/


/saθӘ/	/ɹat∫Ә/	/mira/^1^


/siθӘ/	/ɹit∫Ә/	/rat∫a/


/θit∫Ә/	/ɹimӘ/	/rit∫a/


/θisӘ/	/siɹӘ/	/sira/

*^1^Note that the Spanish nonce-word /mira/, which would be written *mirra*, is distinct from the real Spanish word *mira* /miՐa/ ‘look,’ which is produced with the tap /Ր/. Such minimal pairs contrasting /r/ and /Ր/ exist elsewhere in Spanish; consider *carro* /karo/ ‘car’ vs. *caro* /kaՐo/ ‘expensive’ and *perro* /pero/ ‘dog’ vs. *pero* /peՐo/ ‘but.’*

**Table 2:** Nonce words with language-specific phonetic variants of /l,u/.

/l/		/u/	
English	Spanish	English	Spanish
[t∫aƚsӘ]	[t∫alt∫a]	[t∫ʉt∫Ә]	[t∫uma]
[faƚmӘ]	[filfa]	[fʉt∫Ә]	[fufa]
[hiƚfӘ]	[lafa]	[fʉfӘ]	[fusa]
[ƚit∫Ә]	[lit∫a]	[fʉsӘ]	[mufa]
[ƚifӘ]	[lifa]	[hʉt∫Ә]	[muma]
[maƚfӘ]	[malfa]	[hʉsӘ]	[sut∫a]
[saƚfӘ]	[silma]	[mʉmӘ]	[hut∫a]
[siƚt∫Ә]	[halfa]	[sʉfӘ]	[husa]

#### Stimuli Recordings and Speaker

A single speaker was chosen to record both English and Spanish stimuli, and this was crucial to the experimental task. A single speaker was preferred over recording two monolinguals to avoid voice being a cue to language, and using natural productions of the stimuli ensured there were no acoustic artifacts from splicing or otherwise manipulating segments within a word frame. Using natural productions from a single talker also permitted the selection of the desired segments as target segments, regardless of difficulties isolating them (e.g., with the English /ɹ/).

Since it was also important for the stimuli to lack any language-specific cues, or accent, beyond the controlled target segment, care was taken to recruit a balanced Spanish-English bilingual who produced both languages as natively as possible. The chosen talker was a 37-year-old Spanish-English bilingual who was born and raised in Colombia until the age of 7 at which point he moved to the state of New York with his family. He continued to speak Spanish at home in New York, and as an adult he moved to Texas for graduate school, during part of which he lived in Guatemala and Spain to conduct research. While most of his current daily interactions were in English, he also used Spanish on a daily basis with his family and frequently for translating and interpreting professionally at work. An accentedness rating study was conducted to ensure that the talker’s English and Spanish productions sounded native-like to native English and native Spanish speakers, respectively. In both languages, the talker was rated as native-like as other talkers who grew up as monolingual speakers of each language. See the **Appendix** for a complete description of the accentedness ratings.

The English and Spanish nonce words were recorded in separate sessions to further ensure minimal cross-linguistic transfer. The recordings took place in a sound-attenuated booth using a MOTU UltraLite-mk3 Hybrid recorder at a sampling frequency of 44.1 kHz (16 bit). The talker repeated each nonce word three times so that the clearest repetition could be chosen. The words were written in English and Spanish orthography (e.g., English *leefuh* for [ƚifӘ] and Spanish *chirra* for /t∫ira/) and not in the International Phonetic Alphabet (IPA), so for some items the talker was coached to arrive at the intended pronunciation. The pitch contours were manipulated to match a naturally produced token with a falling contour using Praat (Boersma and Weenink, 2012). The beginning and end points of the F0 contours were set to 170 and 124 Hz to match the values of model token. The intervening pitch points were interpolated between the two end points.

### Participants

Participants (*n* = 53) were recruited through the Department of Linguistics subject pool and received course credit for their participation. To supplement the subject pool participants with the listeners who had the needed language backgrounds, the remaining Spanish–English bilinguals, both early and late (*n* = 27) were recruited through the University of Texas Events Calendar. These participants were paid $10/h for their time.

Listeners completed a language history questionnaire (Chan, 2014) that included questions about participants’ biographical information, the places they had lived and for how long, their language exposure and proficiency, and their language(s) of education. Based on their responses to the questionnaire, participants were divided into three groups: monolingual English speakers with minimal or no exposure to Spanish (Monolingual), Spanish-English bilinguals from the U.S. who acquired both languages in early childhood (Early Bilinguals), and Spanish–English bilinguals from Spanish-speaking countries who acquired English as adults (Late Bilinguals). Participants who did not fit into one of these groups were not included in the final sample (*n* = 24). See **Table 3** for a summary of participant characteristics.

**Table 3:** Demographic information and language background of participants.

	Monolinguals	Early bilinguals	Late bilinguals
N	40	18	22
Mean age	20	20	28
Age range	18–29	18–29	18–43
Females	21	15	11
Mean age (in years) when learned English	0	3.7	10
Mean age (in years) when learned Spanish	12.5	0	0
Mean age (in years) when moved to U.S.	NA	1.3	20.1

Forty participants (21 females) were included in the Monolingual group. All members of this group were from the U.S., had heard English from birth, did not hear another language at home, and were not proficient in any other language. Participants ranged in age between 18 and 29, and the mean age of the group was 20. Of the 40 Monolingual listeners, 24 had studied Spanish in middle and/or high school. One additional participant had some Spanish classes in elementary school, and one further participant reported learning some Spanish as a toddler outside the home. All 26 listeners with some exposure to Spanish reported very low proficiency in the language.

The Early Bilinguals group included 18 participants (15 females) who ranged in age from 18 to 29, with a mean age of 20 years. Eleven of the listeners in the Early Bilinguals group were born and raised in the United States, and the remaining seven participants were born in Mexico (*n* = 6) or Colombia (*n* = 1) and moved to the U.S. before they began elementary school. All listeners in the Early Bilinguals group had learned Spanish at home since birth. Seven participants also learned English at home since birth (four of the U.S.-born participants, three of the foreign-born participants). The remaining 11 participants began learning English when they started elementary school.

Twenty-two listeners (11 females) were categorized as Late Bilinguals since they were born and raised in a Spanish-speaking country and moved to the U.S. after age 14. Listeners in this group ranged in age between 18 and 43, with a mean age of 28 years. Only Late Bilinguals from Latin America participated; listeners from Spain were excluded since /θ/ is phonemic in Peninsular Spanish and the present study included /θ/ as an English-specific phoneme. Listeners were from Mexico (*n* = 11), Argentina (*n* = 2), Peru (*n* = 2), Ecuador (*n* = 2), Bolivia (*n* = 1), Venezuela (*n* = 1), Colombia (*n* = 1), the Dominican Republic (*n* = 1), or some combination of these countries (*n* = 1). Late Bilinguals ranged in the age at which they moved to the U.S. between 14 and 28, with mean age of arrival of 20. All listeners had learned only Spanish at home since birth. Although all had studied English at least informally in school before they moved to the U.S., Spanish was the only language of instruction in both primary and secondary school for all Late Bilingual participants.

### Procedure

Participants completed the nonce-word categorization experiment in the UT Sound Lab in the Department of Linguistics at the University of Texas at Austin. The study was approved by the Institutional Review Board at UT Austin, and the experimenter obtained written informed consent from the participant before beginning the study, in accordance with the IRB’s recommendations. Listeners answered an online language history questionnaire and were tested for normal hearing, followed by the categorization experiment.

Listeners performed the language categorization task in a sound-attenuated booth on a PC running E-Prime 2.0 (Psychology Software Tools, 2010). Listeners wore Sennheiser XX headphones and were oriented to the serial response button box (Psychology Software Tools, 2010). Participants were instructed to place the index and middle fingers of their dominant hand on the two leftmost buttons, which were labeled with “ENG” and “SPAN,” the order of which was counterbalanced across participants. The language that corresponded to each button was also presented on the computer screen, e.g., “ENGLISH” appeared on the left side of the screen for the group of participants who used the left button to indicate English words. Listeners began with a practice block in which they read instructions presented on-screen and decided if each word sounded more like English or more like Spanish. The practice block included 20 real words (10 English, 10 Spanish).

After the practice block, the test portion began. At test, listeners were told they would hear “snippets of speech that were taken out of longer recordings while the speaker was talking in either English or Spanish,” and they were asked to decide if what they heard sounded more like it came from the English recording or the Spanish recording. This wording and context was provided after piloting indicated that some listeners had the impression that they were hearing accented productions instead of words from two languages. To avoid this confusion between accent and language, the categorization task was rephrased to ask about the language being used to produce the word.^6^ Listeners categorized the 56 nonce words (listed in **Tables 1** and **2**) eight times, and stimuli were randomized within each of the eight blocks, for a total of 448 trials. There was a one second pause between a listener’s response and the onset of the audio for the next stimulus. RT was calculated from the onset of the audio file, and categorization decision and RT were recorded for each trial.

## Results

Categorization decision (Spanish or English) and RT were recorded for each trial. Decisions were coded as accurate if words containing the English-specific phoneme /ɹ/ or /θ/ or the English variants [ƚ] or [ʉ] were classified as English and if words with the Spanish-specific phoneme /r/ or the Spanish variants [l] or [u] were classified as Spanish. Trials with the Spanish stimulus *racha* /rat∫a/ and the English stimulus /ɹat∫Ә/ were excluded from the analyses (cf. Nonce Words). RTs were calculated by subtracting the length of the stimulus.wav file from the time calculated by E-Prime between trial onset and button press. This ensured that the RTs analyzed here reflected the length of time for the listener to make a categorization decision, after hearing the end of the stimulus word. Trials with RTs less than 200 ms (*n* = 665; 1.9%) were discarded as spurious responses. RTs were log-transformed from milliseconds to normalize the distribution of responses for the regression analyses. Less than 0.5% of responses exceeded 5000 ms and the distance of these from the mean was reduced in the log transformation. Trials more than three standard deviations above or below a participant’s log-transformed mean were excluded as outliers (*n* = 228; 0.7%). The spurious responses and outliers accounted for 2.6% of all trials (*n* = 893), after *racha* and the English /ɹat∫Ә/ were removed. The following analyses include the remaining 33667 trials (Monolinguals: *n* = 16800; Early Bilinguals: *n* = 7441; Late Bilinguals: *n* = 9426). Accuracy (correct, incorrect) and log-transformed RT were submitted to separate regression analyses, which were analyzed using Bayesian inference with the *glmer2stan* package (v0.995) in R (v3.2.2) to interface with Stan via RStan (v2.8.2).

### Acoustic Analyses

Segmental properties of each stimulus were measured using Praat to ensure that the Spanish and English productions differed in the expected dimensions. The duration and first three formants of both vowels of each stimulus were measured, and the same measures were taken for the /l/ variant in the stimuli containing an English or Spanish /l/. Formant measurements were taken at the vowel midpoint and at 25 and 75% through the vowel. Recall that the vowels /i,a/ were used in the first vowel position of the disyllabic nonce words to create a sufficient number of non-word stimuli, and the second vowel (V_2_) of each nonce word was realized as the full-vowel [a] in Spanish words and as the reduced [Ә] in English words. The Spanish [u] and English [ʉ] segments were target vowels representative of phonetic cues. The acoustic properties of the segments are reported in **Table 4**: in (A) are reported the mean duration and formant values for the English and Spanish productions of the non-target vowels, and in (B) are the measurements of the language-specific variants of the target segments /l,u/. Formant values are the mean of the measurements taken at the midpoint of each vowel. Standard deviations are included in parentheses.

**Table 4:** Acoustic properties of segments.

	Duration (ms)		F1 (Hz)		F2 (Hz)		F3 (Hz)	
	English	Spanish	English	Spanish	English	Spanish	English	Spanish
**(A) Non-target vowels**

/i/	87.0 (22.6)	95.6 (20.3)	369.7 (47.4)	361.0 (31.9)	2245.3 (243.7)	2196.3 (107.9)		
/a/	116.9 (19.0)	99.1 (14.4)	878.8 (67.4)	835.7 (15.1)	1189.4 (74.6)	1524.6 (55.1)		
V_2_	174.4 (29.0)	141.5 (31.4)	693.7 (67.6)	769.8 (130.8)	1367.4 (143.3)	1484.5 (97.7)		

**(B) Target segments**

/l/	63.8 (22.9)	77.7 (17.9)	581.6 (134.7)	383.4 (88.3)	1141.4 (260.3)	1917.4 (331.8)	2999.2 (253.4)	2937.6 (375.9)
/u/	81.7 (11.9)	82.7 (18.3)	415.8 (22.2)	484.5 (170.9)	1560.9 (178.5)	1174.0 (372.5)

In order to test whether the English and Spanish variants were distinct from each other, the concordance statistic (c-statistic) of a logistic regression model was analyzed. The c-statistic is the proportion of outcomes that are correctly predicted by the fitted model. For each vowel, a logistic regression model was constructed in R (RStudio 0.99.489; RStudio Team, 2015) using the *rms* package (v4.2-1) with language (English, Spanish) as the dependent variable and the duration and midpoint measures of F1 and F2 as fixed effects. Measurements were centered and scaled, and duration was removed from the model where singularity remained. The model for English and Spanish /l/ additionally included the midpoint measure of F3 as a fixed effect. Constructing such a model for the c-statistic was preferable to testing for differences between each fixed effect separately since listeners hear the multiple acoustic cues at once; that is, listeners may attend to differences in all three dimensions (F1, F2, and duration), so all three should be considered together when determining if the sounds were distinct in the two languages.

For the two target segments that were measured, /l/ and /u/, it was expected that the formants and the duration of the segment would be sufficient to distinguish the English and Spanish variants. The model with these three main effects as well as the midpoint of F3 made perfect discrimination between the English [ƚ] and the Spanish [l] (*C* = 1.000). For English [ʉ] and Spanish [u], the duration variable was removed to avoid singularity, and the model with the midpoints of F1 and F2 was also highly successful (*C* = 0.969).

The other three segments were the two vowels /i,a/, which were used in the first syllables of the nonce words, and the final vowel of the nonce words. The initial model for /i/, with duration and the midpoint measurements for F1 and F2, produced a c-statistic of 0.681, which represents a moderately good fit to the differences in /i/ in English and Spanish words, but which falls short of the clear distinction between the phonetic variants described above. For /a/ in the position of nucleus of the first syllable, the model was highly successful for discrimination (*C* = 1.000). Finally, the model for the second (unstressed) vowel in the nonce words fit well (*C* = 0.853). The acoustic distance between English and Spanish /a/ in stressed and unstressed positions, as well as those between the /i/ variants, was expected (cf. Bradlow, 1995); see “the discussion” for how the accuracy and RT results should be understood in light of these differences.

### Accuracy Analysis

**FIGURE 1:**
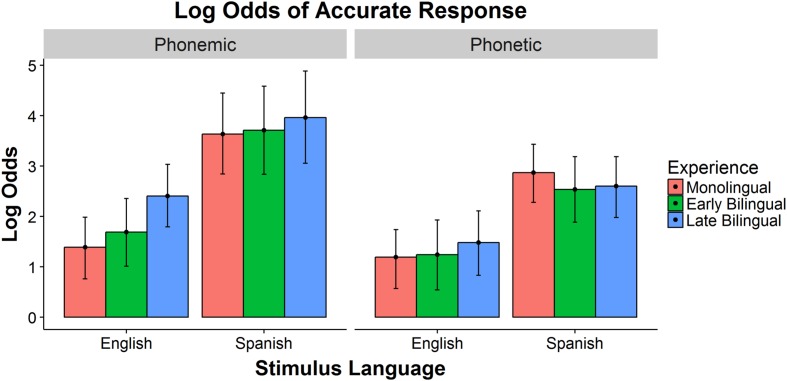
**Predicted log odds of accuracy for phonemic and phonetic cues**.

The mean accuracy score of each group for each stimulus type is presented in **Table 5**. The accuracy results were analyzed using a Bayesian mixed effects logistic regression model with listener language group (Monolingual, Early Bilingual, Late Bilingual), stimulus language (English, Spanish), and stimulus type (phonemic, phonetic) as fixed effects and participant and stimulus word as random intercepts. The models were fitted via a Markov Chain Monte Carlo procedure using STAN (Gelman et al., 2015). Model comparison was performed using the Deviance Information Criterion (DIC; Spiegelhalter et al., 2002). A model with a three-way interaction among the fixed effects provided an improved fit over models with two-way interactions or with only main effects (see **Table 6** for the model summary). The reference group, reflected in the model intercept, represents the accuracy of Monolinguals categorizing stimuli with an English phoneme. The fitted log odds of accuracy for each stimulus language and listener language group are plotted in **Figure 1**, with the phonemic cues in the left panel and the phonetic cues in the right panel. The error bars represent the 95% Bayesian credible intervals.

**Table 5:** Mean accuracy of each listener group for each stimulus type.

		Monolinguals	Early bilinguals	Late bilinguals


English Cues	Phonemic	72.7 (44.5)	78.8 (40.9)	86.1 (34.6)


	Phonetic	70.5 (45.6)	73.2 (44.3)	76.0 (42.7)


Spanish Cues	Phonemic	95.6 (20.5)	96.9 (17.3)	97.6 (15.4)


	Phonetic	91.0 (28.7)	90.4 (29.5)	90.6 (29.1)

*Standard deviations are presented in parentheses*.

**Table 6:** Summary of mixed effects logistic regression model fitting accuracy results.

Predictor	Mean	Posterior *SD*	95% CI	*p*-value
Intercept (Monolingual, English phonemes)	1.391	0.299	(0.763, 1.983)	<0.0001
Phonetic cues	-0.200	0.356	(-0.916, 0.489)	NS
Early bilinguals	0.299	0.273	(-0.244, 0.818)	NS
Late bilinguals	1.014	0.236	(0.546, 1.468)	<0.0001
Spanish cues	2.242	0.459	(1.402, 3.190)	<0.0001
Phonetic ^∗^ Early	-0.247	0.097	(-0.442, -0.059)	NS
Phonetic ^∗^ Late	-0.722	0.097	(-0.911, -0.533)	NS
Phonetic ^∗^ Spanish	-0.562	0.559	(-1.629, 0.521)	<0.0001
Early ^∗^ Spanish	-0.223	0.230	(-0.659, 0.233)	<0.0001
Late ^∗^ Spanish	-0.686	0.231	(-1.135, -0.226)	<0.0001
Phonetic ^∗^ Early ^∗^ Spanish	-0.165	0.256	(-0.674, 0.322)	<0.0001
Phonetic ^∗^ Late ^∗^ Spanish	0.126	0.253	(-0.365, 0.619)	<0.0001

**Random effects**	**Variance**			

Listener	0.892			
Stimulus word	0.970			

#### Comparing Spanish and English Phonemic and Phonetic Cues

Overall, listeners responded more accurately to Spanish cues than to English cues, and to phonemic cues than to phonetic cues. The difference between the languages was greater for phonemic cues than for phonetic cues. The Spanish phoneme was categorized more accurately than the English phonemes (Monolinguals: β = 2.242, posterior *SD* = 0.459, *p* < 0.0001; Early Bilinguals: β = 2.019, posterior *SD* = 0.484, *p* < 0.0001; Late Bilinguals: β = 1.556, posterior *SD* = 0.491, *p* < 0.001), and the Spanish phonetic cues were also categorized more accurately than the English phonetic cues (Monolinguals: β = 1.680, posterior *SD* = 0.367, *p* < 0.0001; Early Bilinguals: β = 1.292, posterior *SD* = 0.373, *p* < 0.001; Late Bilinguals: β = 1.120, posterior *SD* = 0.372, *p* < 0.001). The Early Bilinguals trended toward categorizing the English phonemic cues more accurately than the English phonetic cues (β = 0.448, posterior *SD* = 0.358, *p* = 0.09). The Late Bilinguals categorized English phonemic cues significantly better than English phonetic cues (β = 0.922, posterior *SD* = 0.358, *p* < 0.01). All groups categorized the Spanish phonemic cue more accurately than the Spanish phonetic cue (Monolinguals: β = 0.763, posterior *SD* = 0.451, *p* < 0.01; Early Bilinguals: β = 1.175, posterior *SD* = 0.477, *p* < 0.0001; Late Bilinguals: β = 1.359, posterior *SD* = 0.480, *p* < 0.0001).

#### Comparing Listener Groups

The three listener groups responded very similarly within each segment type, with the exception of the categorization of nonce words with an English phoneme. For the English phonemes, Monolinguals and Early Bilinguals responded less accurately than the Late Bilinguals (vs. Monolinguals: β = 1.014, posterior *SD* = 0.236, *p* < 0.0001; vs. Early Bilinguals: β = 0.715, posterior *SD* = 0.294, *p* < 0.05). There were no group differences in the English phonetic cue conditions, and there were also no significant group differences in response to the Spanish phonemic or the Spanish phonetic cues.

### Reaction Time Analysis

**FIGURE 2:**
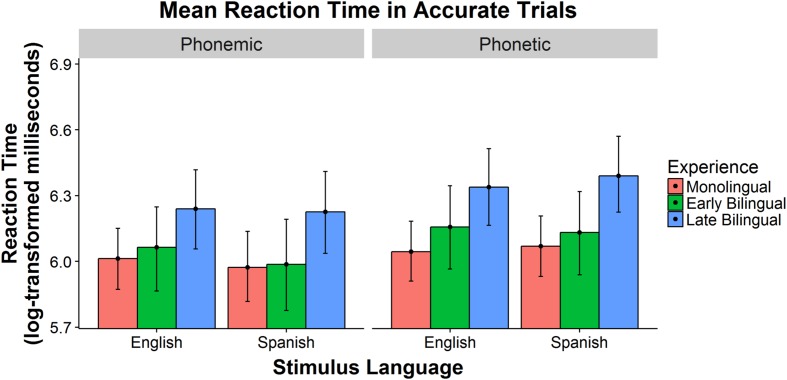
**Model log reaction time for phonemic and phonetic cues in accurate trials**.

The mean RTs (in milliseconds) of each group for correct responses to each stimulus type are presented in **Table 7**. Log-transformed RTs were analyzed using a Bayesian mixed effects linear regression model with listener language group (Monolingual, Early Bilingual, Late Bilingual), stimulus language (English, Spanish), stimulus type (phonemic, phonetic), and accuracy (correct, incorrect) as fixed effects. Participant and stimulus word were included as random intercepts. These models were also fitted via a Markov Chain Monte Carlo procedure using STAN, as described above. Testing for a significant effect of categorization accuracy evaluated the possibility that listeners’ RTs were unaffected by the accuracy of the categorization decision. A model with the same three fixed effects as the accuracy model – listener group, stimulus language, and stimulus type – was significantly improved by adding accuracy as a fixed effect. RTs thus significantly differed between accurate and inaccurate trials, and subsequent models calculated separate betas for each type of trials. The model with a four-way interaction among the fixed effects provided a better fit than models with only main effects, with two-way interactions, or with three-way interactions. See **Table 8** for the model summary. The reference group, reflected in the model intercept, represents the log RT of inaccurate responses by Monolinguals categorizing stimuli with an English phoneme. The fitted log RT for correct responses to each target segment and listener language group are plotted in **Figure 2**. The error bars represent 95% Bayesian credible intervals. The following sections report the results of correct trials from the four-way interaction and the differences between correct and incorrect responses.

**Table 7:** Mean RT (in milliseconds) for correct trials for each listener group and stimulus type.

		Monolinguals	Early bilinguals	Late bilinguals
English cues	Phonemic	542.0 (594.1)	629.8 (727.8)	662.7 (640.7)
	Phonetic	592.3 (742.9)	715.5 (833.4)	770.8 (791.5)
Spanish cues	Phonemic	538.0 (591.8)	530.4 (545.1)	639.8 (675.8)
	Phonetic	595.4 (636.6)	641.2 (711.6)	777.1 (792.7)

**Table 8:** Summary of mixed effects linear regression model fitting log-transformed RT results.

Predictor	Mean	Posterior SD	95% CI	*p*-value
Intercept (Monolingual, English phonemes)	6.191	0.074	(6.046, 6.333)	<0.0001
Phonetic cues	0.040	0.059	(-0.074, 0.158)	NS
Early bilinguals	-0.011	0.114	(-0.236, 0.216)	NS
Late bilinguals	0.359	0.107	(0.146, 0.557)	<0.0001
Spanish cues	0.026	0.102	(-0.179, 0.226)	NS
Correct response	-0.178	0.025	(-0.224, -0.128)	<0.01
Phonetic ^∗^ Early	-0.055	0.053	(-0.155, -0.051)	NS
Phonetic ^∗^ Late	-0.192	0.052	(-0.296, -0.090)	<0.01
Phonetic ^∗^ Spanish	0.037	0.121	(-0.208, 0.273)	<0.10
Early ^∗^ Spanish	0.155	0.152	(-0.145, 0.449)	<0.05
Late ^∗^ Spanish	-0.194	0.153	(-0.492, 0.106)	<0.01
Phonetic ^∗^ Correct	-0.009	0.033	(-0.073, 0.054)	NS
Early ^∗^ Correct	0.062	0.045	(-0.025, 0.150)	<0.10
Late ^∗^ Correct	-0.132	0.046	(-0.223, -0.041)	NS
Spanish ^∗^ Correct	-0.066	0.078	(-0.221, 0.091)	<0.01
Phonetic ^∗^ Early ^∗^ Spanish	0.045	0.164	(-0.265, 0.371)	<0.001
Phonetic ^∗^ Late ^∗^ Spanish	0.389	0.166	(0.058, 0.706)	<0.0001
Phonetic ^∗^ Early ^∗^ Correct	0.116	0.061	(-0.003, 0.237)	NS
Phonetic ^∗^ Late ^∗^ Correct	0.259	0.059	(0.144, 0.378)	<0.05
Phonetic ^∗^ Spanish ^∗^ Correct	0.029	0.088	(-0.150, 0.200)	<0.05
Early ^∗^ Spanish ^∗^ Correct	-0.192	0.156	(-0.499, 0.115)	<0.05
Late ^∗^ Spanish ^∗^ Correct	0.219	0.157	(-0.093, 0.520)	NS
Phonetic ^∗^ Early ^∗^ Spanish ^∗^ Correct	-0.059	0.170	(-0.397, 0.267)	NS
Phonetic ^∗^ Late ^∗^ Spanish ^∗^ Correct	-0.389	0.172	(-0.717, -0.046)	<0.01

**Random effects**	**Variance**			

Listener	0.366			
Stimulus Word	0.151			

#### Comparing Spanish and English Phonemic and Phonetic Cues

For the four cue types, there were few significant differences in RTs. The only differences appeared for the Spanish cues: the Early Bilinguals trended toward faster RTs for the Spanish phonemic cue compared to the Spanish phonetic cues (β = 0.144, posterior *SD* = 0.073, *p* = 0.08), and the Late Bilinguals responded significantly faster to the Spanish phoneme than to the Spanish phonetic cues (β = 0.164, posterior *SD* = 0.073, *p* < 0.05). There was no difference between the Spanish categories for Monolingual listeners. The differences in RT between the English phonemic cues and the English phonetic cues did not reach significance for any listener group. There were also no differences in RTs between the English and Spanish phonemic cues or between the English and Spanish phonetic cues.

#### Comparing Listener Groups

The pattern of differences in RTs among the listener groups was mostly constant across segments: Monolinguals and Early Bilinguals responded with similar RTs, and both these groups were faster than Late Bilinguals. For the Spanish phonemic cue, there was no difference between Monolinguals and Early Bilinguals, and both groups were significantly faster than Late Bilinguals (vs. Monolinguals: β = 0.252, posterior *SD* = 0.100, *p* < 0.01; vs. Early Bilinguals: β = 0.238, posterior *SD* = 0.124, *p* < 0.05). For English phonemes, Monolinguals and Early Bilinguals also responded faster than Late Bilinguals (vs. Monolinguals: β = 0.227, posterior *SD* = 0.100, *p* < 0.01; vs. Early Bilinguals: β = 0.176, posterior *SD* = 0.124, *p* < 0.05), and there was again no difference between the Monolinguals and Early Bilinguals. For trials with Spanish phonetic cues, Monolinguals and Early Bilinguals responded faster than Late Bilinguals (vs. Monolinguals: β = 0.320, posterior *SD* = 0.099, *p* < 0.0001; vs. Early Bilinguals: β = 0.258, posterior *SD* = 0.123, *p* < 0.01), and there was no differences in RTs for the Monolinguals and Early Bilinguals. Finally, for nonce words with an English phonetic cue, Monolinguals and Early Bilinguals were also significantly faster than Late Bilinguals (vs. Monolinguals: β = 0.294, posterior *SD* = 0.100, *p* < 0.0001; vs. Early Bilinguals: β = 0.182, posterior *SD* = 0.123, *p* < 0.05), and Monolinguals trended faster than Early Bilinguals (β = 0.112, posterior *SD* = 0.109, *p* = 0.06).

#### Comparing Accurate and Inaccurate Trials

Overall, RTs for correct responses were faster than for incorrect responses. For Monolinguals, this difference reached significance for all four types of nonce words (English phonemic: β = 0.178, posterior *SD* = 0.25, *p* < 0.01; Spanish phonemic: β = 0.244, posterior *SD* = 0.74, *p* < 0.01; English phonetic: β = 0.187, posterior *SD* = 0.023, *p* < 0.01; Spanish phonetic: β = 0.224, posterior *SD* = 0.035, *p* < 0.01). For Early Bilinguals, correct trials were faster than incorrect trials for the Spanish cues (phonemic: β = 0.374, posterior *SD* = 0.133, *p* < 0.0001; phonetic: β = 0.297, posterior *SD* = 0.052, *p* < 0.001), but there was no difference for the English cues. For Late Bilinguals, the difference between correct and incorrect trials was significant for both kinds of Spanish cues (phonemic: β = 0.157, posterior *SD* = 0.131, *p* < 0.05; phonetic: β = 0.267, posterior *SD* = 0.047, *p* < 0.01) and for the English phonemes (β = 0.310, posterior *SD* = 0.040, *p* < 0.001), but not for the English phonetic cues.

The results of the accuracy and RT analyses are summarized in **Tables 9** and **10**. **Table 9** summarizes how Spanish and English stimuli were categorized by each listener group (A) and how the listeners categorized the different stimuli classes (B). **Table 10** summarizes how the listener groups compared within each stimulus type. The “=” is used to illustrate differences that were not significant, and the “>” and “<” indicate significant differences. The “≫” and “≪” represent differences that approached significance.

**Table 9:** Summary of results from stimuli comparisons.

	Accuracy	Reaction times
**(A) Cross-language comparisons**				

Monolinguals	Spanish phonemic > English phonemic Spanish phonetic > English phonetic	Spanish phonemic = English phonemic Spanish phonetic = English phonetic


Early Bilinguals		
Late Bilinguals		
**(B) Cross-class comparisons**				
Monolinguals	Spanish phonemic > Spanish phonetic English phonemic = English phonetic	Spanish phonemic = Spanish phonetic English phonemic = English phonetic
Early bilinguals	Spanish phonemic > Spanish phonetic English phonemic ≫ English phonetic	Spanish phonemic ≪ Spanish phonetic English phonemic = English phonetic
Late bilinguals	Spanish phonemic > Spanish phonetic English phonemic > English phonetic	Spanish phoneme < Spanish phonetic English phonemic = English phonetic

**Table 10:** Summary of results from listener group comparisons.

	Accuracy	Reaction times
Spanish phonemes	Monolinguals = Early = Late	Monolinguals = Early < Late
English phonemes	Monolinguals = Early < Late	Monolinguals = Early < Late
Spanish phonetic	Monolinguals = Early = Late	Monolinguals = Early < Late
English phonetic	Monolinguals = Early = Late	Monolinguals ≪ Early < Late

## Discussion

The current study tested the sensitivity of monolingual and early and late bilingual adults to language-specific sounds in a nonce-word categorization task to determine which segments listeners are most sensitive to and how language experience influences listeners’ sensitivity. Overall, listeners very accurately categorized phonemic cues and Spanish cues but struggled more with English cues and phonetic cues. There was also a significant interaction between stimulus language and cue type, with the difference between phonemic and phonetic cues greater for Spanish than for English. This difference also significantly interacted with listener group, such that the difference between Spanish and English phonemic cues and Spanish and English phonetic cues was smaller for Late Bilinguals and greater for Early Bilinguals. The categorization accuracy of the Monolinguals, Early Bilinguals, and Late Bilinguals was very similar overall, with the only significant difference between groups occurring for the English phonemic cues, which Late Bilinguals categorized more accurately than the other groups. The response times for Monolingual and Early Bilingual listeners were comparable, and both of these groups responded more quickly than Late Bilinguals for all cue types. Based on models of native and second-language speech perception (Flege, 1987, 1995; Best, 1991), we predicted a greater sensitivity to phonemic properties of lexical and language representations than to phonetic cues. The results here provide new evidence supporting these predictions in a language-decision task with word-length stimuli: early and late bilinguals can use both kinds of segments for categorization, but they were more sensitive to phonemic cues than phonetic cues. Unexpectedly, all listeners were more sensitive to Spanish-specific cues than English-specific cues. Finally, language background had only a limited effect on listeners’ access to these representations.

Overall, there were no differences between the Monolingual and Early Bilingual listeners. The Late Bilinguals were as sensitive to some cues as the other two listener groups, and there was limited evidence that Late Bilinguals might even be more sensitive to some cues. The Late Bilinguals also responded significantly more slowly than the other groups, so it is possible that there was a speed-accuracy trade-off for these listeners; however, it only appeared for the Late Bilinguals’ categorization of English phonemic cues, for which they were significantly more accurate than Monolinguals and Early Bilinguals but also significantly slower. The performance of the Monolinguals and Early Bilinguals reveals that the language representations of the Early Bilinguals, despite their having learned Spanish at home before English, do not differ in the phonemic categories or the phonetic detail encoded in their language representations. This is not to say that our Early Bilinguals would not have shown evidence of their Spanish exposure in other tests, such as production or phoneme identification tasks. The current results do suggest that the ability of Early Bilinguals to generalize about the properties of their native languages and associate phonological properties in particular with each language is not distinct from Monolinguals’ awareness of these language-specific properties. This sets our early Spanish–English bilinguals apart from the early Spanish–Catalan bilinguals in Sebastián-Gallés et al. (2005), whose sensitivity to Catalan-specific contrasts was purportedly compromised by their early exposure to Spanish. Rather, the similarity between our responses from Monolinguals and Early Bilinguals supports the language assessment used by Amengual (2014, 2015), in which adults’ current language exposure and use seem to override the effect of non-simultaneous early exposure and contribute to their equivalent performance (Gertken et al., 2014). The role of ongoing exposure in addition to and even superseding age of acquisition is also supported by Flege and colleagues who found that among listeners with similar ages of acquisition, greater exposure to, use of, and education in the L1 led to less native-like perception and production (Flege, 1991; Flege et al., 1997b; Flege and MacKay, 2004) and grammaticality judgments (Flege et al., 1999b) in the L2. It is important for future work on the association of language and segments to consider dominance and exposure to each language as factors influencing cross-linguistic speech perception in context.

While we only indirectly assessed the bilingual listeners’ language dominance and exposure though the language background questionnaire, the Monolingual and Early Bilingual groups did share some commonalities. Examining those further may assist in understanding the similarities in their categorization decisions and potentially why the Late Bilinguals outperformed these groups in the English phoneme trials. Our Early Bilinguals live and study immersed in their (chronological) L2, English, and as a result, they may have the same awareness of the generalizability of the phonological properties of each of their languages as the monolingual speakers who know only English. The difference between the two bilingual groups for the English phoneme category, on the other hand, may reflect variation in dominance, exposure, or the method of English acquisition. Most of the Early Bilinguals (11 of 18) learned English when they began kindergarten, and language instruction at this age is likely to be much less explicit than the middle and high school foreign-language classrooms in which the Late Bilinguals learned English. Even where there are parallels in L2 teaching at these ages, the experience of English language learning is much more recent for the Late Bilinguals than for the Early Bilinguals, and attending foreign language classes, practicing the language, and laboring to master the rules of and achieve proficiency in the L2 may lead the Late listeners to a greater metalinguistic awareness about properties of the language (Dąbrowska and Street, 2006), including increased sensitivity to language-segment associations. The study of phonological and metalinguistic awareness in adults has been limited to literacy and disorders (e.g., Pennington et al., 1990), although additional work with children has investigated bilingualism (Bruck and Genesee, 1995; Bialystok, 2001) and literacy development (e.g., Anthony and Francis, 2005). It is therefore unclear how metalinguistic awareness and cue sensitivity may affect cross-language speech perception in adults. The current findings suggest that the listeners who acquired an L2 in early childhood may lack the metalinguistic awareness evident in the Late Bilingual listeners, or that this sensitivity may decline into adulthood. Over time and as English proficiency increases, young bilingual listeners may lose their initial phonological sensitivity and may later categorize segments no differently than Monolingual adults who acquired their only language in infancy.

Given the potential differences in language teaching and language learning in kindergarten and high school, the Late Bilinguals may have increased sensitivity to some language-specific phonological properties due to the circumstances of their bilingualism and not necessarily due to the age of acquisition. In fact, this formal training may also explain why there were group differences for the English phonemic cues but not for the English phonetic ones. Phonemic differences across languages may get more attention in foreign-language classes than subsegmental differences between categories shared by the two languages. Just as the phonetic cues were more difficult for listeners in general, Late Bilinguals may not have had the same metalinguistic instruction about English phonetic differences and so may have been less able to associate those cues with English, even though this was possible for the phonemic cues. Future work on cue sensitivity should work to separate recency of language acquisition from method of language acquisition to disentangle how these factors influence phonological awareness and especially awareness of subsegmental differences. For example, Early Bilinguals may be more sensitive to English phonemic cues during earlier stages of English acquisition, and we might also expect listeners who acquire a language without formal classes (e.g., from being immersed in a new community) to be less sensitive to language-specific cues, especially phonemes, than listeners who study the language in a formal setting.

The consistency of categorization accuracy across the three listener groups suggests that language experience was less important than cue salience in this task. Phonemic cues were more accurately categorized than phonetic cues, for both English and Spanish, supporting the parallel distinction made between new and similar phones in Flege (1987, 1995)’s Speech Learning Model (SLM). In this model, second language learners create independent categories for sounds judged to be “new” (unique to the L2 and not present in the L1), which facilitates the production and perception of such sounds. Phones that are recognized as similar to existing L1 segments are discriminated less well if no new category is established for them. The phonemes in the present task may be like the SLM’s new phones, even for the Monolinguals who have not acquired Spanish, and as such they are immediately recognizable as language-specific sounds (Best, 1991), which leads to more accurate categorization. In contrast, the phonetic cues pattern like the SLM’s similar phones, a category for which, according to Best (1991), the L2 or non-dominant language sounds would be mapped to the L1 or dominant-language categories. This would cause more competition in deciding between English or Spanish for the language identity of the word.

There may have also been an effect of the specific segments included in each category. Since there was only one Spanish-specific phonemic cue included, the Spanish phoneme category in fact represents listener responses to a single sound, the Spanish trill /r/, which was easily perceived and strongly associated with Spanish phonology for all three listener groups. The English phoneme category may have been very different in this sense, since it included the English rhotic /ɹ/ and the interdental fricative /θ/. Fricatives and interdentals in particular are acquired late by English-learning children (Clark, 2003; Dodd et al., 2003), and even native-English-speaking adults are susceptible to mishearing /θ/ more than they mishear other segments (Cutler et al., 2004). That is, there may be inherent differences in the perceptual salience of the two English phonemes, irrespective of the strengths of associations between English and each segment. Since only a single Spanish phonemic cue was available and given the asymmetry in salience of the English phonemic cues, future work should more systematically compare a wider range of phonemes in other language pairs to consider whether there may be variability within the phonemic category. However, despite the inherent difficulty of at least the English /θ/, it is even more striking that the Late Bilinguals outperformed the groups that had acquired the English phonemes in childhood. In fact, since the Late Bilinguals may be aware of /θ/ being a phonemic sound in Peninsular Spanish, we might have expected this awareness to cause confusion and thus fewer accurate responses in English phoneme trials for the Late Bilinguals, but just the opposite was the case. This suggests that the absence of this phoneme in the native language and dialects of the Late Bilinguals may have heightened their sensitivity to /θ/. Instead, the difficulty all listeners had responding to the English phoneme category may be motivated by perceptual salience more generally, and future work should further probe variation with each of these cue types.

The difficulty listeners from all backgrounds experienced in accurately categorizing phonetic cues also requires further investigation. The English [ƚ] is more velarized, i.e., produced with the tongue further back in the oral cavity, than the Spanish [l], while the English [ʉ] is fronted, so the difference between English and Spanish phonetic cues is unlikely to be due to a single property that sets English apart from Spanish, since the English variants differ in opposite directions from the Spanish ones. It may be that listeners hear more variation in English input between lighter or darker /l/ and more or less fronted /u/ across dialects, speakers, and phonological contexts than exists for Spanish [l] and [u]. However, it would be surprising if our monolingual English listeners were also sensitive to the greater consistency of these segments in Spanish, given their lack of exposure to the language.^7^ Furthermore, if the variability present in the realization of these sounds in English motivated the difference in accuracy between English and Spanish segments, we should expect a different categorization pattern entirely. A light [l] or a backed [u] may be either from Spanish or English, since these variants exist in many dialects of English, so the Spanish phonetic cues should have received responses more mixed between the languages. It is the darker [ƚ] and fronted [ʉ] that should be unambiguously associated with English, but in fact we find the English cues receive more of a mix of Spanish and English categorization decisions while the Spanish cues are relatively consistently identified as Spanish.

While every effort was made to create nonce words that were equally plausible in both languages, except for the language-specific target segment, the naturally produced stimuli used here inevitably carried additional indicators of language. The phonotactic restrictions of Spanish may have meant that the CVCV stimuli were simply more Spanish-like than English-like, even though this word structure is permitted in English. The Spanish-ness of these stimuli is supported by the reactions of participants in two pilot studies; in the first pilot, theoretically congruous stimuli that overlapped English and Spanish in all segments, e.g., /t∫ima/, were categorized as Spanish significantly more than English, and in the second pilot (cf. Procedure), listeners reported confusion about whether words were English or English-accented Spanish. In the present study, listeners from all three language backgrounds were able to overcome this potential bias toward Spanish for English: the log odds of responding correctly were significantly above 0 (chance performance) in all four cases, including for the English segments. Therefore, listeners showed sensitivity to the English-ness of the English cues even if the word structure is less common in English than it is in Spanish. Furthermore, Monolinguals might not be expected to suffer from such a potential bias as much as the bilingual groups, since the Monolinguals do not have representations of Spanish phonotactics against which to judge the nonce word forms. Instead, their categorization patterns were in line with the bilingual groups’. Why, then, might listeners have been less accurate in categorizing stimuli with English cues?

The difficulties that persisted for English cues are especially interesting given that the naturally produced nonce words used here likely contained multiple phonetic cues to language. As was mentioned in the discussion of nonce words, the disyllabic nature of the nonce words meant that the unstressed vowel /a/ in the second syllable was reduced to [Ә] in the English words; therefore, all the English nonce words contained both a language-specific target segment (e.g., /ɹ/) and the reduced vowel. Furthermore, the acoustic analyses of the /i/ and /a/ vowels in the first syllable of the nonce words indicate that there were also language-specific differences in the productions of these non-target segment (cf. Acoustic Analyses). But again, despite these potential additional cues to language, listeners categorized the English-specific segments less accurately than Spanish cues. Given the more accurate performance of the Late Bilinguals than the other groups for English phonemes we might be tempted to conclude that the Late Bilinguals were better able to use these supplementary language-specific cues than their peers, but their accuracy did not significantly differ from the Monolinguals and Early Bilinguals in the English phonetic condition. If the Late Bilinguals were more sensitive to the English-ness of the nonce word filler vowels in the phonemic condition, where they outperformed their peers, it is unclear why they wouldn’t have been able to make use of the additional cues in the English phonetic words.

Moving forward, it will continue to be important to consider the contributions of language-specific segments in the context of a word, as discussed earlier, since listeners may use different processing strategies and respond to the same sound categories differently when presented in isolation and in context. To this end, it will be necessary to also involve language pairs for which there are more language-specific contrasts and a wider variety of segments to be studied than those available for English and Spanish. All phonemic cues used here were consonants, with a necessary but confounding overreliance on the differences in rhotics across the languages. Similarly, the mispronunciation studies in Spanish and Catalan by Sebastián-Gallés et al. (2005) and Amengual (2014, 2015) were restricted in scope, and focused only on vowels. Contrasting a language pair that differs more significantly in both consonants and vowels at the phonemic and phonetic levels would provide the evidence needed to further test the conclusions drawn from the present results.

Finally, the current study speaks to other related speech perception phenomena, namely foreign-accent detection. To date, our knowledge of the perception of foreign-accented speech has been largely based on monolingual listeners, but the findings of the present study support the inclusion of listeners actually proficient in, and not just familiar with, the L1 of the accented speech. Based on our results, bilingual listeners might be expected to identify accented talkers as well as monolingual listeners, and if the foreign accent contains non-native phonemic cues like those tested here, late bilinguals might be more sensitive to accented speech than other listeners. Benefits of exposure to accented speech have likewise been reported for categorizing sentences produced in regional (Clopper and Pisoni, 2004, 2007) and foreign (Vieru et al., 2011) accents. High-exposure listeners also processed foreign-accented words faster and more accurately than low-exposure listeners (Witteman et al., 2013), so listeners with experience can attend to the relatively few cues available in a single word. Even so, given the nature of the naturally produced words and sentences used in these studies, it is not clear what cues the listeners with greater exposure were using in their processing, or which cues the less-experienced listeners were not able to capitalize on. We might expect foreign-accented speech to contain more of the difficult phonetic cues that most challenged our Monolingual listeners, and this could explain the performance of the low-familiarity listeners in Vieru et al. (2011) and Witteman et al. (2013). The contribution of phonemic and phonetic cues to foreign-accented speech detection could be tested by controlling these cues in real words, as was done in the present study with nonce words, to determine if real foreign-accented words with deviant phonemic cues are in fact categorized more easily than words with phonetic cues. Furthermore, the processing of foreign-accented speech may also be influenced by the presence of phonemic and phonetic cues. Since phonetic cues are less clearly linked to a specific language and listeners of all backgrounds are less sensitive to deviations in phonetic cues, speech that contains only phonetic deviations (e.g., from more proficient L2 speakers) may be easier to process than speech that also contains phonemic deviations.

In summary, the results of the nonce-word categorization task indicate that listeners are better able to use Spanish-specific cues than English-specific cues and that listeners categorize phonemic cues, modeled on Flege’s (1987, 1995) “new” sounds, better than phonetic cues. This distinction supports similar divisions made between native and non-native sounds in speech perception literature more generally and for second language acquisition in particular (Flege, 1987, 1995; Best, 1991). Our findings also show similarities in categorization patterns across listener groups, in parallel with the work of Mack (1989) and Flege et al. (1999a) on early bilinguals’ phoneme discrimination, and even the late bilinguals categorized the nonce-word stimuli like early learners. The early bilinguals’ sensitivity to English-specific cues was not degraded by their early exposure to and proficiency in Spanish, deviating from the conclusions of Sebastián-Gallés et al. (2005), but their knowledge of Spanish also did not improve the accuracy of their language classification decisions for Spanish nonce words, which might have been expected given the advantages for high-exposure listeners in accent categorization tasks (e.g., Witteman et al., 2013). Such facilitation was observed for the late bilinguals for words with English phonemic cues, although the late bilingual listeners responded significantly more slowly than the other groups for all cues. The study of additional language pairs will strengthen the conclusions we make here about differences in listener sensitivity to language-specific phonemic and phonetic cues by providing additional segments and contrasts and allowing for systematic comparisons, e.g., of consonantal and vowel contributions to each category. The finding that listeners use phonemic cues more successfully than phonetic cues in word contexts should shape future directions of work on the perception of foreign-accented speech and cross-language speech perception.

## Author Contributions

CPB, CB, and RS jointly designed the project. CPB and RS designed the stimuli, and CPB and CB worked together to analyze the data. All three authors were responsible for interpreting the results. CPB wrote the manuscript draft, and CB and RS added considerable critical commentary and revisions. All three have approved this manuscript and are accountable for all aspects of the work regarding accuracy and integrity.

## Conflict of Interest Statement

The authors declare that the research was conducted in the absence of any commercial or financial relationships that could be construed as a potential conflict of interest.

## Footnotes

1 See Carlson et al. (2015) for recent work on early bilinguals’ use of L1 phonotactics in speech perception.

2 While the Speech Learning Model distinguishes between new and similar phones in a second language, this binary may not be sufficient to include all relationships between the sounds of one’s native language and the categories in a second language. For example, it is unclear how to classify a shared phone with different statuses in each language, e.g., both Spanish and English use the tap [Ր], but this sound is phonemic in Spanish and allophonic in English.

3 In fact, our results suggest that late bilingual listeners were even more sensitive than the other listener groups to the association of /θ/ with English. See the discussion for additional analysis of how the different listener groups categorized stimuli with /θ/.

4 While vowels can be described as differing from one another along (minimally) three continuous dimensions (F1, F2, and duration), there can in fact be phonemic or “new” categories across languages. This would be the case, for example, for English listeners perceiving French /y/, which does not exist as a category in English, even though it may initially be confused with English /u/ or French /u/ (Flege, 1987); English listeners treat French /y/ as a language-specific category sooner than they recognize French /u/ as a category unique from English /u/. This, however, is not the case for any Spanish-specific vowel, which are in line with the French /u/-English /u/ relationship.

5 The phoneme identified here as /h/ is alternately realized as /x/ in some dialects of Spanish (Hualde, 2005). The speaker chosen to record the stimuli uses /h/ in his dialect of Spanish; see “Stimuli Recordings and Speaker.”

6 This phrasing invites the possibility that listeners may have looked for other patterns in the stimuli to make their categorization decisions, such as the appearance of language-specific morphemes in the nonce words. All nonce words did end in /a/, which is the Spanish morpheme for feminine adjectives (e.g., *rojo* /roho/ ‘red-MASC’ vs. *roja* /roha/ ‘red-FEM’) and is also one of the morphemes for third-person singular (e.g., *habla* /abla/ ‘speaks-3SG’). However, since all nonce words uniformly ended in /a/, it is not a feature that distinguishes some stimuli from others. See discussion for potential language-specific properties of the nonce words.

7 We would additionally have to assume that exposure to Spanish-accented English is sufficient for the development of phonological categories that accurately reflect the properties of these categories as they are realized in Spanish.

## Appendix

To ensure that the stimuli talker’s productions were native-like in both languages, an accentedness rating study was completed. Native English and native Spanish listeners rated the nativeness of the productions of eight talkers, including the stimuli talker. All talkers recorded Æsop’s *The North Wind and the Sun* in Spanish and English, and the final set of talkers included one male and one female from each of the following four groups: monolingual English talkers, L1 English talkers who learned Spanish late and had completed college and graduate coursework in Spanish, L1 Spanish talkers from Latin America who learned English late and had moved to the U.S. to attend college, and early Spanish-English bilinguals (including the stimuli talker). The recordings from these eight talkers were divided into seven phrases, yielding 56 sound files of the talkers’ English and 56 sound files of their Spanish.

The raters included ten monolingual English listeners and 10 L1 Spanish listeners from Latin America who learned English after age 14. None participated in the main study. Raters heard productions in their native language and decided how native- or foreign-sounding each production was by using the mouse to click on a horizontal line. The line appeared on the screen after the audio presentation of each sentence and represented a continuum between “Perfectly native sounding” (labeled as such at the left extreme) and “Very foreign sounding” (so labeled at the right extreme). The Spanish translations “Suena totalmente nativo” and “No suena nada nativo” were used in the Spanish version with the native Spanish listeners and the talkers’ Spanish productions. The accentedness rating was recorded as the x-intercept of the mouse at the click. The 56 sentences were randomized for each listener.

Accentedness ratings were converted to *z*-scores to account for listeners using the continua differently, and the *z*-transformed accentedness ratings for English and Spanish productions were submitted to separate mixed-effects linear regression models using the *lme4* (v1.1-7) and *lmerTest* (v2.0-20) packages in R (RStudio 0.99.489; RStudio Team, 2015). Listener was included as a random intercept, and testing talker as a fixed effect significantly improved the fit of a model with the random intercept alone, for both the English model (χ^2^ = 1317.3, df = 7, *p* < 0.001) and the Spanish model (χ^2^ = 948.25, df = 7, *p* < 0.001). See **Table A1** for the model summaries. The stimuli talker (early bilingual male) was designated as the referent class for the talker variable. The intercept for the stimuli talker was significantly less than zero (*p* < 0.001) in both the English and Spanish models and was thus significantly closer to the “Perfectly native sounding” extreme than to the center for both languages. The stimuli talker’s English was not rated as significantly different from the monolingual English male (*p* = 0.29) or the L1 English male (*p* = 0.12), and he was rated as significantly more native sounding than all other talkers (at least *p* < 0.01) except the monolingual English female (*p* < 0.05)^8^. The stimuli talker’s Spanish was also rated as significantly more native sounding than all the other talkers (*p* < 0.001), except for the L1 Spanish male and female, with whom there was no significant difference in rating (for L1 Spanish male, *p* = 0.80; for L1 Spanish female, *p* = 0.29).

**Table A1:** Model summaries for mixed-effects linear regression models predicting accentedness ratings.

Predictor	Estimate	Standard error	*t*-value	*p*-value
**(A) English productions**				
Intercept (Stimuli talker)	-0.632	0.037	-17.186	<0.001
Monolingual male	0.0554	0.052	1.065	0.29
Monolingual female	-0.131	0.052	-2.516	<0.05
L1 English male	-0.082	0.052	-1.575	0.12
L1 English female	0.163	0.052	3.127	<0.01
Early bilingual female	0.613	0.052	11.785	<0.001
L1 Spanish male	2.123	0.052	40.797	<0.001
L1 Spanish female	2.318	0.052	44.537	<0.01

**Random effects**	**Variance**			

Listener	<0.001			
Residual	0.095			

**(B) Spanish productions**				
Intercept (Stimuli talker)	-0.873	0.051	-17.062	<0.001
Monolingual male	2.272	0.072	31.404	<0.001
Monolingual female	2.241	0.072	30.970	<0.001
L1 English male	1.292	0.072	17.861	<0.001
L1 English female	0.661	0.072	9.144	<0.001
Early bilingual female	0.458	0.072	6.323	<0.001
L1 Spanish male	-0.018	0.072	-0.255	0.80
L1 Spanish female	0.077	0.072	1.070	0.29

**Random effects**	**Variance**			

Listener	<0.001			
Residual	0.183			
